# Research Priorities for Diabetic Ketoacidosis: An Evidence and Gap Mapping Review

**DOI:** 10.3390/medsci13020053

**Published:** 2025-05-01

**Authors:** Nicolas Sieben, Mahesh Ramanan

**Affiliations:** 1 Intensive Care Services, Mater Public Hospital, Brisbane, QLD 4101, Australia; nicolas.sieben@mater.org.au; 2 Intensive Care Services, Royal Brisbane and Women’s Hospital, Metro North Hospital and Health Services, Brisbane, QLD 4029, Australia; 3 Intensive Care Unit, Caboolture Hospital, Metro North Hospital and Health Services, Brisbane, QLD 4510, Australia; 4 Faculty of Health, Queensland University of Technology, Brisbane, QLD 4000, Australia; 5 The George Institute for Global Health, University of New South Wales, Sydney, NSW 2052, Australia

**Keywords:** evidence gap, evidence gap mapping, diabetes, diabetic ketoacidosis, renal medicine, intensive care

## Abstract

Background/Objectives: Diabetic ketoacidosis (DKA) is a common acute complication of diabetes with treatment consisting of reversal of cause, insulin administration, fluid resuscitation and electrolyte repletion. Yet, many aspects of DKA management are currently based on low-quality evidence or physiological rationale. This evidence and gap map review presents an overview of the current body of literature and identifies evidence gaps in relation to therapeutic interventions for DKA. Methods: Interventions and outcomes relevant to DKA were identified and iteratively developed to produce a coding model for the proposed evidence and gap map. PubMed was searched with Me SH terms relevant to the identified interventions and outcomes. Studies identified were screened and assigned interventions and outcomes. Interventional research was uploaded to EPPI-Reviewer and EPPI-Mapper to produce the evidence and gap map. Results: The search identified 1131 studies, of which 18 were non-human and 345 were duplicates. A total of 768 unique studies were screened, and 118 were identified as interventions (52 pediatric and 66 adult studies). A total of 26 high-quality studies, 88 medium-quality studies and 4 low-quality studies were identified. These 118 studies were coded into the proposed DKA evidence and gap map. The intervention domains were fluid therapy, insulin therapy, electrolyte replacement, adjunct therapies and admission type. The outcome domains were DKA resolution, insulin duration, length of stay, morbidity and mortality, complications, and biochemical parameters. Conclusions: Fluid type and insulin infusion administration were prominent in the current literature. These studies frequently used DKA resolution and complications associated with DKA such as electrolyte disturbances and cerebral edema as the primary outcomes. Substantial gaps were identified with scant evidence to guide prophylactic electrolyte administration, enteral intake and adjunctive therapy (thiamine, bicarbonate). Even for well-investigated interventions such as fluids and insulin, substantial gaps existed, particularly for patient-centered and healthcare service outcomes.

## 1. Introduction

Diabetic ketoacidosis (DKA) is common complication of diabetes, often with severe presentations and significant morbidity and mortality [1]. Most DKA episodes are precipitated by a group of common triggers including infections, missed insulin doses and other concurrent critical illness during intensive care admissions [2,3]. DKA occurs when the balance of endogenous insulin and glucagon is disturbed, through absolute or relative insulin deficiency and glucagon excess, promoting ketone body generation, which leads to severe raised anion gap metabolic acidosis [4]. Prolonged hyperglycemia and ketosis produce an osmotic diuresis resulting in severe dehydration or hypovolemic shock [4]. The cornerstones of DKA management are reversal of cause, large-volume crystalloid replacement (typically normal saline (NS, 0.9% Sodium Chloride)), intravenous insulin infusion and electrolyte repletion. Adjunctive therapy may include sodium bicarbonate, thiamine, mechanical ventilation and renal replacement therapy, among others [5,6].

Various components of DKA management have recently been investigated [4,7]. However, a sparse amount of literature exists that evaluates DKA interventions outside of fluid volume administration and insulin replacement characteristics. Few DKA studies have attempted to evaluate the effect of interventions on clinical outcomes, DKA resolution and biochemical response [8,9,10].

New research techniques including evidence and gap mapping (EGM) may be used to summarize the state of the published literature on a particular topic and assist in identifying and characterizing evidence gaps [11]. This may assist researchers in designing and developing future research. We undertook this study to summarize current evidence and identify research gaps in DKA management.

## 2. Methods

The EGM for DKA was developed in an iterative process beginning with a structured review of common DKA pathways currently used [11]. Common groups of interventions were thematically organized into groups for consideration of inclusion into the EGM. Physician opinion and structured searches were used to augment the intervention pool for EGM analysis. A similar process was used for measurable outcomes commonly seen with DKA admissions and treatment pathways. The above process identified interventions including fluids (type, volume and/or rate, one- or two-bag systems, and hemodynamic or biochemical targets), insulin (bolus, infusion rate and timing of subcutaneous insulin), electrolyte replacement (oral vs. intravenous [IV], prophylactic and targets), enteral intake (timing, dose and type), other adjuncts (bicarbonate and thiamine administration) or selective admission (ward and Intensive Care Unit [ICU] admission). Outcomes that were identified with this process included the following: DKA resolution (American Diabetic Association [ADA] definition, Bahrain Diabetes Society [BDS] definition or other definition), intravenous insulin duration, length of stay (Emergency Department [ED], hospital and ICU), morbidity and mortality (mortality or days alive and hospital-free), health care costs, complications (hyperchloremia, hypoglycemia, hypokalemia, hypophosphatemia, cerebral edema or other complications) and biochemical response (lactate, bicarbonate, ketones/glucose, pH and other biochemical parameters).

Studies for inclusion were identified in an iterative process based on the above interventions and outcomes. PubMed was searched with an iterative combinatoric Me SH search for DKA [Me SH term] with each identified intervention and outcome between the years 2014 and 2024. Superseding Me SH terms were used when appropriate. After initial searching, interventions and outcomes were rationalized once 10% of the initially identified studies were screened, and additional searches were added for any additional identified outcomes or interventions. Each identified study was cataloged and coded with the above EGM intervention–outcome coding scheme. After all studies were screened, all interventional studies were imported to EPPI-Reviewer (https://eppi.ioe.ac.uk/cms/Default.aspx?tabid=2914 (accessed on 20 April 2025)) and converted to a JSON export compatible with mapping in EPPI-Mapper (https://eppi.ioe.ac.uk/cms/Default.aspx?tabid=3790 (accessed on 7 February 2025)) [12]. The DKA EGM was produced, the literature was further classified into adult or pediatric studies, and the level of evidence was provided. (High quality: clinical trial, systematic review/meta-analysis or randomized control trial. Medium quality: cohort study, comparative study, cross-sectional study, observational study, prospective study or retrospective study. Low quality: review article, protocol, modeling study, letter or case report).

## 3. Results

**Figure 1:**
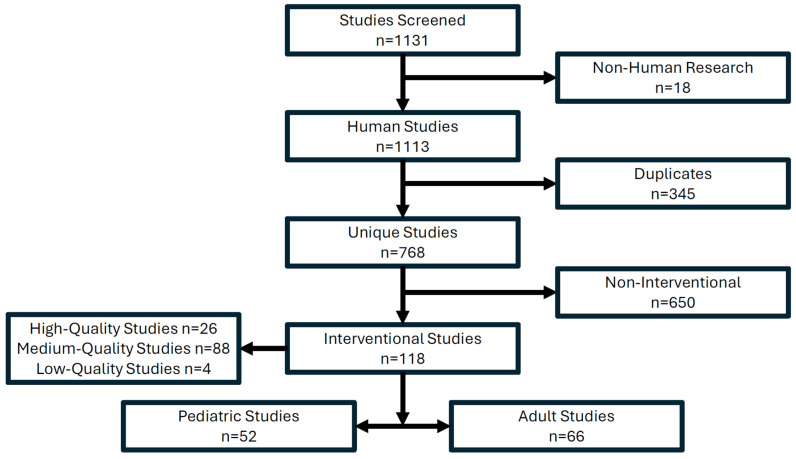
PubMed search results identifying interventional research for the DKA EGM.

The search results yielded 1131 results across all PubMed MeSH searches. After screening, 18 were excluded as non-human trials and 345 were duplicates, leaving 768 unique studies (Figure 1). Further refinement showed only 118 of the remaining 768 studies were interventional, representing 15.4% of all studies passing screening (Figure 1). Of the 118 studies, 52 were pediatric and 66 were adult studies (Figure 1). A total of 26 high-quality studies (mostly systematic reviews and meta-analysis), 88 medium-quality studies (namely, retrospective, prospective and observational studies) and 4 low-quality studies were identified among the interventional studies (Figure 1). All 118 studies, which passed screening and had a codable intervention, were included in the DKA EGM (Supplementary Materials).

The two most populated interventional areas of evidence include fluid administration and insulin infusions [7,13]. Fluid choice and volume/rate of fluid administration were evaluated in multiple studies with high-quality evidence. Volume of fluid use was often studied and strongly supported in multiple high-quality studies. Outcomes such as biochemical parameters and complications in fluid administration studies (type and volume) were consistently studied as they are key monitored variables in most emergency departments and inpatient DKA protocols. High-quality studies evaluating balanced crystalloids versus normal saline were abundant, including two systematic reviews with meta-analysis and ten randomized control studies. Fifteen medium- quality studies, including twelve retrospective studies, complemented the detected systematic reviews and randomized control trials. Complications such as cerebral oedema, hypoglycemia and hypokalemia are common monitoring targets in both fluid- and insulin infusion- focused studies [14,15]. Critical illness indicators such as lactate and acute kidney injury (classified as “complication: other”) were occasionally observed in fluid- and insulin infusion- focused studies [16,17]. The duration of DKA was often studied in the context of dose of insulin infusion and the resuscitation fluid type [18,19]. Two randomized control trials, one systematic review with meta-analysis and a singular meta-analysis, provided high-quality evidence in the evaluation of insulin infusion rates. High- quality insulin infusion studies were complemented by a further sixteen retrospective studies. The association of the rate of insulin infusion and timing of subcutaneous insulin with fluid resuscitation type and the duration of intravenous insulin infusion was frequent across the detected studies. Comparable outcomes such as complications, length of stay or biochemical parameters in fluid-focused studies were seen in studies evaluating insulin infusion rate. Other endpoints in both fluid trials and insulin infusion studies included mortality and length of stay in hospital [20,21].

Multiple evidence gaps were detected in the resulting DKA EGM (Supplementary Materials). Insulin boluses and fluid resuscitation based upon hemodynamic targets were infrequently studied. Two-bag fluid systems were uncommonly seen in the EGM, but the amount of evidence is likely to increase after the completion of ongoing trials specifically looking at a two-bag system [22]. Interestingly, fluid resuscitation targets based on vitals were not reported in any case and not considered as an endpoint for fluid resuscitation. Evidence regarding electrolyte replacement, including route of administration, prophylactic administration and specific targets, were not often reported as a primary intervention. Electrolyte concentrations were a commonly reported outcome measure.

The current literature infrequently comments on adjunctive bicarbonate administration or prophylactic thiamine, and both remain under-investigated adjuncts in DKA management [23,24]. DKA definitions were commonly defined by a set of biochemical parameters usually including pH, bicarbonate level and/or serum ketones and not by a formal DKA definition and thus are underreported in the EGM. Health care costs were infrequently reported, and days alive and hospital-free were rarely reported. The length of stay in hospital was well documented except for ED length of stay, which likely has become amalgamated with the overall hospital admission. Most complications were well reported except for hypophosphatemia.

## 4. Discussion

This study has summarized a wide body of literature pertaining to DKA management. The two most prominently investigated interventions were fluid and insulin administration. Fluid resuscitation in DKA presentations has previously been centered around NS as the preferred fluid [25]. Multiple studies have challenged the typical crystalloid (normal saline) against balanced crystalloids such as Hartmann’s or plasmalyte-148 [25]. The literature suggests balanced crystalloids offer a less acidotic fluid replacement to exacerbate ongoing ketoacidosis, but this remains disputed in clinical practice [26,27].

A two-bag fluid resuscitation system has also been postulated in the literature to reduce the hyperchloremic effect, but further studies are required. Hemodynamic-based targets offer a new endpoint for the hypovolemia seen in DKA, but no studies have addressed its utility [28]. Within the observed fluid trials, few studies have commented on the health care-associated costs, commented on days alive and hospital-free, or used a consistent DKA definition preferring a set of biochemical parameters. Perfusion and critical illness indicators such as lactate and hypophosphatemia were rarely reported, while common DKA complications such as hypoglycemia and hypokalemia were. The debate on balance crystalloid versus normal saline fluid resuscitation types require further evidence, while hemodynamic-target-based, two-bag resuscitation systems and critical illness endpoints such as lactate and hypophosphatemia offer areas of potential investigation.

Insulin administration was another frequently observed intervention in the EGM. The most frequent intervention included that of the rate of insulin infusion, with insulin boluses and timing of subcutaneous insulin administration being witnessed much less frequently. Studies monitoring the effect of a given insulin infusion rate were frequently correlated with duration of insulin infusion, hospital or intensive care length of stay, and complications such as hypoglycemia, hypokalemia and cerebral edema [29,30]. Serum bicarbonate, ketones and pH were also frequently monitored in insulin infusion rate studies. Days alive and hospital-free, health care costs, hypophosphatemia and serum lactate were often omitted from insulin infusion studies, suggesting a valuable endpoint for investigation. Few studies included an insulin bolus before infusion with endpoints like isolated insulin infusion studies [31]. Subcutaneous insulin administration had greater representation in the literature, suggesting a more readily accepted intervention, often the endpoint of DKA admissions. Endpoints in subcutaneous insulin administration studies include both insulin infusion studies and insulin bolus studies [30]. Insulin bolus studies and administration of subcutaneous insulin are under-represented in the literature and variably used in the clinical setting of DKA presentations, opting for the standardized insulin infusion in many DKA protocols. Further studies on early subcutaneous insulin and initial bolus of insulin represent potential areas of future studies while their usefulness remains tenuous.

Multiple interventions appeared under-represented in the EGM, including general ward versus intensive care admissions, electrolyte replacement, enteral feeding and treatment adjuncts including infused sodium bicarbonate and thiamine. The observed studies were sparse except for those on general ward versus intensive care admissions and renal replacement therapy, which had far fewer studies than fluid or insulin infusion trials. Intensive care admission was observed to improve insulin infusion duration and common complications such as hypoglycemia and readily measurable biochemistry such as serum bicarbonate, ketones, pH and lactate [32,33]. The measured biochemical parameters are likely due to the availability of blood gas analyzers and renal replacement therapy frequently cited in these studies [34]. Intensive care admission studies also suggest greater severity in the DKA presentations captured as patients would need to meet ICU admission criteria. Intention ward admission was infrequently seen and had similarly observed endpoints compared to intentional ICU admission. Both ward and intensive care admissions ignored emergency department lengths of stay and did not use a consistent DKA definition. It is well known that there is increased monitoring in intensive care, but due to resource demands, this represents a difficult avenue for future research and a lack of general ward applicability [32]. Intensive care admission does, however, suggest an avenue for close monitoring for future intervention investigation.

Targeted electrolyte replacement in DKA was infrequently observed in the included studies. Many studies and clinical consensus acknowledge the complications of insulin infusions, particularly hypoglycemia and hypoka lemia. The few included studies involving electrolyte replacement evaluated specific electrolyte targets throughout management or gave prophylactic replacements for correlation with their desired endpoints. Electrolyte-related complications were frequently monitored but represent an area of investigation that aims to limit their morbidity or improve recovery via improved electrolyte management [35]. The endpoints in studies monitoring electrolyte administration were varied, and the entire group of coded endpoints should be investigated.

Enteral feeding was a hypothesized intervention in DKA management, but evidence was very limited. Clinically, enteral feeding is often associated with insulin infusion to subcutaneous insulin transition [36]. Enteral feeding is challenging for patients in DKA, but enteral feeding has been shown to directly help to reverse ketoacidosis through stimulating endogenous insulin release [36]. Studies involving enteral feeding were rare, and the measured endpoints in those studies were scattered across the codable endpoints of the EGM. Early enteral feeding is an avenue of intervention that largely has not been investigated but is clinically useful in transitioning insulin regimes. The association between enteral feeding and various DKA endpoints remains a notable intervention for future studies.

Adjuncts in DKA management were also considered. Thiamine and bicarbonate infusions are uncommon adjuncts in DKA protocols. Thiamine is a cofactor in the catabolism of sugars and, when deficient, greatly impairs the utilization of sugars within the cell and increases cellular resistance to insulin, worsening the DKA [37,38]. Low-quality evidence was observed in thiamine studies. Thiamine administration is a low-risk medication with a theoretical benefit for DKA and glucose utilization at the cellular level. Thiamine administration has not been definitively studied, but it may be a rapidly deployable, potential adjunct for DKA management. Bicarbonate infusions were also considered as an infrequent DKA intervention. Bicarbonate solutions are an alkalotic adjunct to reverse acidosis but come at the cost of excessive CO_2_ production, which can precipitate type 2 respiratory failure in a hyperventilating DKA patient [24,25]. There is varied thought on the risk–benefit ratio of bicarbonate administration, and thus, few studies are observed to examine its effect [39]. The existing studies involving bicarbonate administration observe the duration of insulin infusion, length of stay, hypoglycemia rates, hypokalemia rates and common biochemical endpoints, including serum lactate, pH, ketones and serum bicarbonate. Despite bicarbonate infusions being a controversial and reserved adjunct in DKA management, further research can be considered in DKA presentations. A plethora of DKA interventions is observed in the literature, with various interventions and outcomes ready to be investigated.

EGM is an emerging review technique with multiple benefits, but EGM also has its limitations. EGM is a novel review technique that identifies research abundance and scarcity, comparing the previous literature surrounding the outcomes of select interventions. Novel intervention–outcome pairing can now be seen in the body of DKA research. EGM is typically not comparable to the structure of a scoping or systematic review and thus is limited by its unstructured search. It is also inherently limited by the quality and quantity of literature available on the topic. EGM, however, takes a snapshot of what is known and, even if a select study is missing, should not drastically alter the density of research in a given intervention–outcome pairing.

## 5. Conclusions

The current DKA literature provides some guidance on fluid administration and insulin infusion parameters, but much remains to be investigated in order to optimize DKA management. Hemodynamic targets, enteral feeding, electrolyte replacement, DKA adjuncts, and metabolic monitoring remain promising avenues of intervention investigation. Patient-centered outcomes including mortality and days alive and hospital-free; biochemical outcomes including serum ketones, serum lactate and serum phosphate levels; and healthcare outcomes including health care costs and days alive and hospital-free remain under-investigated across many previously well-studied interventions.

## Data Availability

No new data were created or analyzed in this study.

## Supplementary Materials

The following supporting information can be downloaded at https://www.mdpi.com/article/10.3390/medsci13020053/s1, Supplementary File S1: Search Strategy Details A search of PubMed was conducted ending in December 2024. Years included in this study were 2014–2024. No language, country- of- origin or other filters were used.

## Abbreviations

The following abbreviations are used in this manuscript:

ADA	American Diabetes Association
BDS	Bahrain Diabetes Society
DKA	Diabetic Ketoacidosis
DM	Diabetes Mellitus
EGM	Evidence and Gap Map
IV	Intravenous
NS	Normal Saline
