# Supplementary material for: Research Priorities for Diabetic Ketoacidosis: An Evidence and Gap Mapping Review
Source: Med Sci (Basel). 2025 May 1;13(2):53. doi: 10.3390/medsci13020053 (PMC12101225; doi:10.3390/medsci13020053)
Supplement: Supplementary file 1 [file medsci-13-00053-s001.zip › medsci-3490774-supplementary.html]

EPPI-Mapper


X

X Accessibility

Web accessibility refers to the practice of designing and developing websites and web-based applications in a
way that makes them usable and accessible to all individuals, including those with disabilities. This includes
ensuring that web content can be easily understood, navigated, and interacted with by people with a wide range
of abilities and disabilities, including those who use assistive technologies such as screen readers.

This Eppi Mapper generated map conforms to the World Wide Web Consortium's Web Content Accessibility
Guidelines (W3C WCAG) Version
2.1 Level AA to the maximum extent possible. These guidelines explain how to make web content more accessible
for people with disabilities. The features in this map include:

- If the default settings have been used when generating this map, the overall colour design has been set to
  a more visually contrasting appearance.
- Links in the application have been converted to buttons to accommodate screen readers.
- Aria status updates now inform screen readers when modals open and selected records change.
- Tab focus is contained within opened modals.
- The style selection functionality has been moved to the top menu for easier access.
- A new style, Textual view, has been added to the map. This view presents the record counts within each
  node in a text readable format.
- URL links for individual records have been made descriptive.
- The map generation tool will inform the user if the selected background and font colour contrast meet
  accessibility requirements.

  

**Limitations**

At the present time, while the keyboard focus does stay within each opened modal, the focus cannot be moved
to individual checkboxes or radio buttons or, in the View Records modal, to all fields in the full record.
This means that those items, along with the nodes in the map, can only be selected using a mouse or pointing
device. In future updates we hope to address this limitation.

Filters


Hide Headers
Show Headers


Fullscreen
Exit Fullscreen


About


Submit a Study


View Records


Accessibility

Generated using v.2.3.0 of the EPPI-Mapper
powered by EPPI Reviewer
and created with


by the
Digital Solution Foundry team.
